# Gastro oesophageal reflux disease (GORD)-related symptoms and its association with mood and anxiety disorders and psychological symptomology: a population-based study in women

**DOI:** 10.1186/1471-244X-13-194

**Published:** 2013-07-24

**Authors:** Livia Sanna, Amanda L Stuart, Michael Berk, Julie A Pasco, Paolo Girardi, Lana J Williams

**Affiliations:** 1 Unit of Psychiatry, Neurosciences, Mental Health and Sensory Organs Department (NeSMOS), Faculty of Medicine and Psychology, Sant’Andrea Hospital, Sapienza University of Rome, Rome, Italy; 2 IMPACT Strategic Research Centre, School of Medicine, Deakin University, P.O. Box 281, Geelong 3220, Australia; 3 Department of Psychiatry, The University of Melbourne, Parkville, Australia; 4 Orygen Youth Health Research Centre, Parkville, Australia; 5 Florey Institute for Neuroscience and Mental Health, The University of Melbourne, Parkville, Australia; 6 NorthWest Academic Centre, Department of Medicine, The University of Melbourne, Western Health, St Albans, Australia; 7 Department of Medicine, Barwon Health, Geelong, Australia

**Keywords:** Mood disorder, Anxiety disorder, Gastro-oesophageal reflux disease (GORD), Psychological symptoms, Comorbidity, Depression, Gastrointestinal tract, Somatic, Comorbidity

## Abstract

**Background:**

Psychopathology seems to play a role in reflux pathogenesis and vice versa, yet few population-based studies have systematically investigated the association between gastro-oesophageal reflux disease (GORD) and psychopathology. We thus aimed to investigate the relationship between GORD-related symptoms and psychological symptomatology, as well as clinically diagnosed mood and anxiety disorders in a randomly selected, population-based sample of adult women.

**Methods:**

This study examined data collected from 1084 women aged 20-93 yr participating in the Geelong Osteoporosis Study. Mood and anxiety disorders were identified using the Structured Clinical Interview for DSM-IV-TR Research Version, Non-patient edition (SCID-I/NP), and psychological symptomatology was assessed using the General Health Questionnaire (GHQ-12). GORD-related symptoms were self-reported and confirmed by medication use where possible and lifestyle factors were documented.

**Results:**

Current psychological symptomatology and mood disorder were associated with increased odds of concurrent GORD-related symptoms (adjusted OR 2.1, 95% CI 1.3-3.5, and OR 3.0, 95% CI 1.7-5.6, respectively). Current anxiety disorder also tended to be associated with increased odds of current GORD-related symptoms (p = 0.1). Lifetime mood disorder was associated with a 1.6-fold increased odds of lifetime GORD-related symptoms (adjusted OR 1.6, 95% CI 1.1-2.4) and lifetime anxiety disorder was associated with a 4-fold increased odds of lifetime GORD-related symptoms in obese but not non-obese participants (obese, age-adjusted OR 4.0, 95% CI 1.8-9.0).

**Conclusions:**

These results indicate that psychological symptomatology, mood and anxiety disorders are positively associated with GORD-related symptoms. Acknowledging this common comorbidity may facilitate recognition and treatment, and opens new questions as to the pathways and mechanisms of the association.

## Background

Researchers have repeatedly hypothesized that psychopathology plays a role in reflux pathogenesis and vice versa [1-4]. Gastro-oesophageal reflux disease (GORD) is a common condition due to stomach content flowing back through the lower oesophageal sphincter (LOS), causing bothersome symptomatology characterized by acid regurgitation and heartburn [5]. Prevalence figures vary from 20% of the general population in the United States and the United Kingdom to 5% in China, with Australian studies reporting a prevalence of 9.2% [6,7].

While there is an extensive evidence base suggesting an association between irritable bowel syndrome and psychiatric disorders [8], associations with GORD are relatively poorly researched. GORD and mental well-being have been examined in both gastrointestinal and psychiatric-based clinical care settings with reports of increased likelihood of GORD amongst depressed individuals [9,10] and increased risk of mental illness including depression and anxiety [11], neuroticism [12] and psychological distress [2,3,13] amongst GORD sufferers. Furthermore, frequency and duration of GORD symptoms [2] and a poorer response to treatment including surgical intervention [14] and proton pump inhibitor therapy [4,5], have also been linked to mental well-being. On the other hand, Kamolz and colleagues demonstrated improvements in mental health related quality of life following surgery for GORD [15].

Few population-based studies have investigated the association between GORD-related symptoms and psychopathology [16]. Those that have, report increased odds of reflux in people suffering anxiety and depression, although most utilising self-report symptom scales [1,17,18] and one study using data from medical records [10]. We have previously described increased odds of GORD-related symptoms in men with mood and anxiety disorders in the Geelong Osteoporosis Study (GOS) male cohort, using the Structured Clinical Interview for DSM-IV-TR Research Version, Non-patient edition (SCID-I/NP) [19], thus providing a rationale for this study. Encouraged by the need for population-based evidence, we aimed to investigate the relationship between GORD-related symptoms and psychological symptomatology, as well as clinically diagnosed mood and anxiety disorders in a randomly selected, population-based sample of adult women.

## Methods

### Participants

The Geelong Osteoporosis Study (GOS) is an age-stratified random population-based sample of women recruited from the Commonwealth of Australia electoral rolls [20]. Participants were recruited from the Barwon Statistical Division between 1994 and 1997, with an additional sample of 20–29 year olds recruited between 2004 and 2008, allowing for continuing investigation of the full adult age range. From a pool of 1127 women who participated in the GOS 10 year follow-up assessment, 32 who did not undergo the psychiatric assessment and 11 who did complete the medical history section were excluded from the current analyses, resulting in a final sample of 1,084 women aged 20-93 yr. Written informed consent was obtained from all participants and the study was approved by the Barwon Health Human Research Ethics Committee.

### Assessments

Data from the Structured Clinical Interview for DSM-IV-TR Research Version, Non-patient edition (SCID-I/NP) [21] was used to determine the age of onset of mood and anxiety disorders. All psychiatric interviews were conducted by trained personnel with qualifications in psychology. Major depressive disorder, bipolar disorder (types I and II), dysthymia, minor depression, mood disorder due to medical condition and substance induced mood disorder were collectively termed mood disorder. Anxiety disorder was defined as the presence of panic disorder, agoraphobia, social phobia, specific phobia, obsessive compulsive disorder, generalised anxiety disorder, anxiety disorder due to a medical condition, substance-induced anxiety disorder or anxiety disorder not otherwise specified.

Psychological symptomatology was assessed using the General Health Questionnaire (GHQ-12) [22], a validated screening tool designed to detect non-psychotic psychiatric disorders in community settings, based on assessment of current state. Binary scoring was used for the 12 items, yielding total scores ranging from 0 to 12, and a cut-off of >3 was applied to define caseness indicative of mental health dysfunction [22,23].

Current medication use was documented. The presence of GORD was self-reported and/or identified by use of medication taken daily for hyperacidity, reflux or ulcers. From a list of common medical conditions, participants were asked, at interview, if they had ever experienced GORD or GORD-related symptoms (lifetime yes/no) and whether the condition had been present during the previous 12 months (current yes/no). Height was measured to the nearest 0.1 cm and body weight to the nearest 0.1 kg. Body mass index (BMI) was calculated as weight/height^2^ (kg/m^2^) [24]. Alcohol consumption (average grams consumed daily over a 12 month period) was estimated using a validated food frequency questionnaire [25]. Women were classed as active if they participated in light to vigorous activity on a regular basis. Tobacco smoking was classified as current or not. Socio-economic status (SES) was determined using Socio-Economic Index For Areas (SEIFA) scores based on the 2006 Australian Bureau of Statistics Census data [26]. These values were used to derive an Index of Relative Socioeconomic Advantage/Disadvantage (IRSAD), which accounts for high and low income, educational attainment, type of occupation, and other measures of wealth (such as owning a car, or number of bedrooms in a dwelling) and then divided into quintiles.

### Statistical analysis

Statistical analyses were performed using Minitab (version 16; Minitab, State College, PA). Values of p < 0.05 were accepted as significant. Differences in characteristics between the groups were compared using chi-square analyses and Mann–Whitney. Binary logistic regression techniques were used to evaluate the relationship between current GORD-related symptoms and current mood/anxiety disorders and psychological symptomatology, and lifetime GORD-related symptoms and lifetime mood/anxiety disorders. Models were adjusted for age, alcohol consumption, BMI, SES, physical activity and smoking status where appropriate and interactions between exposure variables were examined.

## Results

Characteristics of the whole group (n = 1084) are shown in Table 1. Of this group, 311 women (28.7%) had a lifetime history of mood disorder [major depressive disorder - 244 (78.5%), dysthymia - 19 (6.1%), minor depression - 31 (10.1%) and bipolar disorder - 23 (7.4%)] and 91 were identified with current mood disorder [major depressive disorder - 48 (52.7%), dysthymia - 19 (20.8%), minor depression - 13 (14.3%) and bipolar disorder - 14 (15.4%)]. A total of 144 participants (13.3%) had a lifetime history of anxiety disorder [generalised anxiety disorder – 10 (6.9%), panic disorder - 59 (41.0%), agoraphobia – 8 (5.6%), social phobia - 23 (16.0%), specific phobia - 36 (25.0%), obsessive compulsive disorder 14 (9.7%), anxiety due to a general medical condition - 2 (1.4%), substance-induced anxiety disorder - 3 (2.1%) and anxiety disorder not otherwise specified - 17 (11.8%)] and 83 were identified with current anxiety [generalised anxiety disorder – 10 (12.0%), panic disorder - 14 (16.9%), agoraphobia – 6 (7.2%), social phobia - 10 (12.0%), specific phobia - 32 (38.6%), obsessive compulsive disorder 6 (7.2%), anxiety due to a general medical condition - 1 (1.2%), substance-induced anxiety disorder - 2 (2.4%) and anxiety disorder not otherwise specified - 12 (14.5%)].

**Table 1:** Characteristics for the whole group and women with or without current and lifetime GORD

	**All**	Current GORD-related symptoms*			**Lifetime GORD-related symptoms**		
		**Yes**	**No**	**P value**	**Yes**	**No**	**P value**
	**n = 1084**	**n = 114**	**n = 958**		**n = 151**	**n = 933**	
**Age at interview (yr)**	50.7(34.3, 65.9)	68.6 (58.4, 77.4)	48.1 (32.1, 63.7)	<0.001	66.1 (56.6,77.3)	47.8 (31.5, 63.2)	<0.001
**BMI (kg/m**^**2**^)*	26.3 (23.4, 55.0)	28.6 (25.0, 33.9)	26.0 (23.2, 30.5)	<0.001	28.3 (24.5, 33.6)	26.0 (23.3, 30.5)	<0.001
Alcohol consumption (g/day)*	2.9 (0.3, 12.0)	0.8 (0.1, 11.0)	3.1 (0.4, 12.2)	0.18	1.5 (0.0, 9.9)	3.2 (0.4, 12.4)	0.2
Smokers (current)*	153 (14.1%)	12 (10.5%)	139 (14.5%)	0.25	16 (10.6%)	137 (14.7%)	0.18
Physically active*	840 (77.6%)	77 (67.5%)	758 (79.3%)	0.004	98 (64.9%)	742 (79.7%)	<0.001
**Socio-economic status**							
**Quintile 1 (most disadvantaged)**	167 (15.4%)	21 (18.4%)	145 (15.1%)	0.79	27 (17.9%)	140 (15.0%)	0.85
**Quintile 2**	232 (21.4%)	25 (21.9%)	206 (21.5%)		32 (21.2%)	200 (21.4%)	
**Quintile 3**	253 (23.3%)	28 (24.6%)	221 (23.1%)		37 (24.5%)	216 (23.2%)	
**Quintile 4**	211 (19.5%)	18 (15.6%)	188 (19.6%)		26 (17.2%)	185 (19.8%)	
**Quintile 5**	221 (20.4%)	22 (19.3%)	198 (20.7%)		29 (19.2%)	120 (20.6%)	
**Mood disorder (current)**	114 (10.6%)	18 (15.8%)	72 (7.5%)	0.003	-	-	-
**Anxiety disorder (current)**	83 (7.7%)	11 (9.7%)	70 (7.3%)	0.37	-	-	-
Current symptomatology (GHQ >3)*	161 (15.2%)	27 (25%)	129 (13.8%)	0.002	-	-	-
**Mood disorder (lifetime)**	311 (28.7%)	-	-	-	47 (31.1%)	264 (28.3%)	0.48
**Anxiety disorder (lifetime)**	144 (13.3%)	-	-	-	26 (17.2%)	118 (12.7%)	0.13

*Missing values: BMI n = 29, alcohol consumption n = 33, physically active n = 2, smokers n = 1, current GORD n = 12, GHQ n = 27.

Values are given as median (IQR) or n (%).

Women with current GORD-related symptoms were older, had a higher BMI, were less likely to be physically active and were more likely to report psychological symptomatology and have a current mood disorder; otherwise the groups were similar (Table 1). Age- (model I) and age- and BMI- adjusted (model II) odds ratios for women with current GORD-related symptoms and mood/anxiety disorders/symptomatology is shown in Table 2. Those with current psychological symptomatology had increased odds of concurrent GORD-related symptoms (adjusted OR 2.1, 95% CI 1.3-3.5, p = 0.005). Current mood disorders were associated with a 3-fold increased odds of current GORD-related symptoms (adjusted OR 3.0, 95% CI 1.7-5.6, p < 0.001) and current anxiety disorder tended to be associated (adjusted OR 1.8, 95% CI 0.9-3.8, p = 0.10).

**Table 2:** Age-adjusted (model I) and age- and BMI- adjusted (model II) odds ratios for GORD in women with mood and anxiety disorders and symptomatology

	**Model**	**GORD-related symptoms - current**		
		**Odds ratio**	**95% CI**	**P value**
**Symptomatology (current)**	**I**	2.2	(1.3 – 3.6)	0.003
	**II**	2.1	(1.3 – 3.5)	0.005
**Mood disorder (current)**	**I**	3.1	(1.7 – 5.6)	<0.001
	**II**	3.0	(1.7 – 5.6)	<0.001
**Anxiety disorder (current)**	**I**	1.8	(0.9 – 3.7)	0.10
	**II**	1.8	(0.9 – 3.8)	0.10
	**Model**	**GORD-related symptoms - lifetime**		
		**Odds ratio**	**95% CI**	**P value**
**Mood disorder (lifetime)**	**I**	1.6	(1.1 – 2.5)	0.02
	**II**	1.6	(1.1 – 2.4)	0.02
**Anxiety disorder (lifetime)**	**I- BMI <30 kg/m**^**2**^	1.2	(0.6 – 2.4)	0.64
	**I- BMI >30 kg/m**^**2**^	4.0	(1.8 – 9.0)	0.001

Those with a past history of GORD-related symptoms were older, had a higher BMI, consumed less alcohol and were less likely to be physically active than those with no past history; otherwise the groups were similar (Table 1). A lifetime history of mood disorders was associated with a 1.6-fold increased odds of lifetime GORD-related symptoms (adjusted OR 1.6, 95% CI 1.1-2.4, p = 0.03; Table 2). BMI was an effect modifier in the relationship between lifetime anxiety disorders and lifetime GORD-related symptoms. Among obese women, lifetime anxiety disorders were associated with a 4-fold increased odds of lifetime GORD-related symptoms (age-adjusted OR 4.0, 95% CI 1.8-9.0, p = 0.001). There was no association between lifetime anxiety disorders and GORD-related symptoms in non-obese women.

## Discussion

This cross-sectional, population-based study showed a strong association between psychological symptoms, mood disorders and GORD-related symptoms in adult women. The relationship between anxiety disorders and GORD-related symptoms was not as consistent; a positive trend was observed when comparing current anxiety disorders and current GORD-related symptoms, however an association between lifetime anxiety and GORD-related symptoms was only evident among obese women.

Our findings are consistent with previously reported community-based studies demonstrating a relationship between GORD and psychological symptomatology and mood/anxiety disorders [1,10,17,18]. In a Norwegian study (HUNT 2) of over 40,500 participants, those diagnosed with reflux, defined by the presence of either severe symptoms of recurrent heartburn or acid regurgitation, showed an increased likelihood of anxiety and depressive symptoms, as measured by the Hospital Anxiety and Depression Scale, compare to those without reflux [1]. Similarly, another large-scale study [10] demonstrated a 72% increase odds of GORD amongst those with depression in a sample of over 40,000 individuals.

Among women with a lifetime history of anxiety disorder in our sample, only participants affected by obesity were at increased risk of GORD-related symptoms. Findings from Wiklund et al. [4] may help explain these results; BMI was shown to be associated with endoscopy-negative reflux and patients affected by this type of reflux (functional heartburn) were more likely to have anxiety than those with endoscopy-positive acid reflux-symptoms [5]. Thus our positive association between anxiety and self-reported GORD-related symptoms in participants with obesity may be due to them suffering from functional heartburn, rather than GORD. The self-reported diagnosis of GORD-related symptoms in our sample does not allow clarification of this issue.

There are many possible explanations for the association between psychopathology and GORD-related symptoms. As suggested by Kamolz and Velonovich [16], the relationship may be attributed to changes in oesophageal motility and LOS function in response to stressors. In addition, visceral hypersensitivity, which would also explain the frequent overlap between heartburn and irritable bowel syndrome [27] may be caused by psychological factors influencing stimuli processing in the central nervous system (CNS) [28]. Although little is known about the morphological organization of serotonergic neurons in the oesophagus, laboratory and clinical investigations have indicated that serotonin, the main target of both depression and anxiety treatment, plays a role in oesophageal motility [29,30], leading to neurohormonal interaction between the CNS and the gastrointestinal system. On the other hand, it is plausible that the presence of GORD-related symptoms can evoke feelings of depression or anxiety [16] or that there are a subset of patients with co-occurring vulnerability to both mood/anxiety and reflux symptoms [3]. Lastly, there is no evidence, endoscopic or otherwise, that identifies which factors of reflux are significantly related to mental illness [2]. Therefore, future research should focus on factors that mediate the relationship between psychological conditions and GORD, helping to pinpoint the mechanism of action.

The comorbidity between psychological illness and GORD has been shown to be clinically relevant, in that the response to GORD treatment, both pharmacological [5] and surgical [14], is lower amongst patients with psychopathology, and certain antidepressants, especially tricyclics, appear to aggravate GORD symptoms [10]. On the other hand, studies have shown the efficacy of medications used to treat depression and anxiety, such as trazodone [31] and citalopram [32], in improving oesophageal symptoms. Thus, recognising the common comorbidity amongst GORD and mood/anxiety disorders may facilitate recognition, and aid treatment choices. Furthermore, Nunez-Rodriguez [2] reported that GORD was associated with other psychological symptom patterns according to the Symptom CheckList-90-R, suggesting the relationship between psychological factors and reflux may extend beyond mood and anxiety disorders.

Methodological limitations of the current study include the cross-sectional design, which precludes temporal and causal speculation, self-reported GORD diagnosis and the lack of severity evaluation of GORD-related symptoms. Current diagnostic criteria for heartburn, based on endoscopic and pH-metric findings, symptomatology and response to proton pump inhibitor therapy, distinguish between erosive reflux disease, non-erosive reflux disease and functional heartburn. Despite this differentiation, Lee and colleagues [5] found depression not to differentiate in prevalence among the three groups, whilst anxiety tended to be more prevalent in the functional heartburn group. Furthermore, the confounding role of somatisation, frequently associated to depression and anxiety, and previously shown to be related to heartburn symptomatology [2] was not examined. It is also possible that GORD-related symptoms experienced during pregnancy may have been included in the lifetime history. However, as participants were not assessed during pregnancy, it is unlikely that current GORD-symptoms were pregnancy-related. Strengths of the current study include the population-based design and combination of the two types of psychological assessment; a reference standardized semi-structured diagnostic assessment (SCID-I/NP) and a self-report questionnaire (GHQ-12). Most of the literature is based only on self-report questionnaire [4,16], with findings being extrapolated to reflect psychiatric diagnoses, whereas we were able to investigate sub-threshold psychopathology as well as diagnosed psychiatric disorders.

## Conclusions

These results provide population-based evidence that psychological symptomatology, mood and anxiety disorders are positively associated with GORD-related symptoms. Further research dissecting the pathways and mechanisms of this association is warranted. In the meantime, taking a holistic approach to treatment of these conditions is likely to improve symptoms, treatment choices and prove cost-effective.

## Abbreviations

BMI: Body mass index; CNS: Central nervous system; CI: Confidence Intervals; GORD: Gastro oesophageal reflux disease; GOS: Geelong Osteoporosis Study; GHQ-12: General Health Questionnaire; IRSAD: Index of relative socioeconomic advantage/disadvantage; IQR: Interquartile range; LOS: Lower oesophageal sphincter; OR: Odds ratio; SEIFA: Socio-economic index for areas; SES: Socio-economic status; SCID-I/NP: Structured clinical interview for DSM-IV-TR research version, non-patient edition; DSM-IV-TR: Diagnostic and Statistical Manual of Mental Disorders, 4th edition, text revision.

## Competing interests

Livia Sanna, Amanda L Stuart and Mark A Kotowicz have no conflicts of interest, including specific financial interests and relationships and affiliations relevant to the subject matter or materials discussed in the manuscript.

Paolo Girardi has in the past three years received research support from Lilly and Janssen, participated in advisory boards for Lilly, Organon, Pfizer, and Schering, and received honoraria from Lilly and Organon.

Julie A Pasco has received speaker fees from Amgen, Eli Lilly and Sanofi-Aventis and funding from the Geelong Region Medical Research Foundation, Barwon Health, Perpetual Trustees, the Dairy Research and Development Corporation, The University of Melbourne, the Ronald Geoffrey Arnott Foundation, ANZ Charitable Trust, the American Society for Bone and Mineral Research, Amgen (Europe) GmBH and the NHMRC.

Michael Berk has received Grant/Research Support from the NIH, Simons Foundation, CRC for Mental Health, Stanley Medical Research Institute, MBF, NHMRC, Beyond Blue, Geelong Medical Research Foundation, Bristol Myers Squibb, Eli Lilly, Glaxo SmithKline, Organon, Novartis, Mayne Pharma, Servier and Astra Zeneca. He has been a paid consultant for Astra Zeneca, Bristol Myers Squibb, Eli Lilly, Glaxo SmithKline, Janssen Cilag, Lundbeck and Pfizer and a paid speaker for Astra Zeneca, Bristol Myers Squibb, Eli Lilly, Glaxo SmithKline, Janssen Cilag, Lundbeck, Organon, Pfizer, Sanofi Synthelabo, Solvay and Wyeth.

Lana J Williams has received Grant/Research support from Eli Lilly, Pfizer, The University of Melbourne, Deakin University and the NHMRC.

## Authors’ contributions

LS and ALS took part in the conception and design of the study, acquisition of the data, data cleaning and statistical analysis, interpretation of the analysis and took primary responsibility for writing the manuscript. MB, JAP and MAK took part in the conception and design of the study, interpretation of the analysis and critically revised the manuscript. PG took part in the interpretation of data and critically revised the manuscript. LJW took part in the conception and design of the study, interpretation of the analysis, critically revised and supervised the writing of the manuscript. All authors read and approved the final manuscript.

## Pre-publication history

The pre-publication history for this paper can be accessed here:

http://www.biomedcentral.com/1471-244X/13/194/prepub
